# Supplementary material for: Chemical composition, seasonal variability and source identification of PM2.5 in an urban background site in Medellín—Colombia
Source: PLoS One. 2026 May 27;21(5):e0350267. doi: 10.1371/journal.pone.0350267 (PMC13215534; doi:10.1371/journal.pone.0350267)
Supplement: Attachment — Submitted filename: Reviewer3-PONE-D-25-58360.pdf [file pone.0350267.s002.pdf]

The manuscript entitled “Chemical Composition, Seasonal Variability, and Source Identification of PM2.5 at an Urban Background Site in Medellín – Colombia” analysed a year-long concentration of PM2.5 and its sources in Colombia. Even though the manuscript is written in a proper manner, it requires some significant changes that need to be implemented before publication. So a major revision is required to improve the quality.

General Comments

1. In many places, authors used very generic statements which need to be changed with scientific terminology. This needs to be addressed throughout the manuscript.
2. In the introduction, the Flow of the contents needs to be re-examined. A few statements are provided that contradict other statements within the introduction that need to be addressed.
3. The introduction should cover how this study will be useful further for researchers or policy makers and what new information it provides to the existing scientific knowledge.
4. Section 2.1 should include information on the local sources, such as industries, transport corridors, etc, that surround the study area.
5. Section 2.2 has a lot of abbreviation which needs to be abbreviated in the first place and abbreviations used consequently in the manuscript.
6. Sections 2.3.1 to 2.3.3 can be reduced to a single paragraph, just mentioning the methodology and the procedure followed, which can be referred to some previous studies that they conducted, and can be mentioned here with reference instead of explaining the procedure again.
7. The results section seems to be weak with more rigorous scientific discussions. Authors should provide more analysis to illustrate the differences in concentration between the wet 1, wet 2, dry 1, and dry 2 seasons. Interannual changes.
8. The authors should look into the different means and sum concentrations mentioned in the manuscript. There are numerous mismatches in mean and sum values throughout the manuscript.
9. Figures 2, and 3,4 are not properly labelled. Only unit is written. It represents what, like mass concentration, etc., and what is count? This needs to be updated.
10. The discussion sections need significant improvement. The section is written with redundant sentences and the information is not scientifically discussed.

Specific Comments

1. Line 32, the authors used generic statements instead of specific scientific terminology, such as “within cities”, which can be replaced with “local sources”
2. Similarly, line 33- emission generated miles away as “long range transportation of sources”
3. Line 34 PM2.5 is referred to as aerosols. In most cases, aerosols are referred to as

smaller particles.

4. Line 41-42 remain there – deposited.
5. Line 42. The sentence contradicts the previous statement.
6. Lines 60 to 70 can be revised to provide the basic information about the study area with previous studies' results instead of CCN, because CCN impacts are more significant with particles in the smaller size range.
7. Lines 78 to 79 provide information about how the concentration increases over the period of time in Medellin.
8. Line no. 95 check one temperature unit.
9. Lines 98 to 100 do not match the information provided in lines 180. Cross-check and correct.
10. If possible, replace Figure 1 with an image which shows the surrounding Ares road network instead of the DEM map.
11. Line no 115, the authors should clarify whether the filter papers were desiccated before and after the measurement for moisture removal. The moisture content plays a major role in chemical composition of the collected sample. If not preserved properly, the composition of measured pollutants will change.
12. Line no 120 mentions 112 samples were collected and in table 1 the sum of the samples collected is 110 this needs to be corrected.
13. Inconsistent verb tenses. Use PAST TENSE ONLY: Line no. 128. Then, the samples are stored at -20 ± 3 °C until chemical characterisation.
14. Line no. 142: The concentration of water-soluble ions (K+, Na+, Mg2+, Ca2+, SO42-, NO3 -, F- and Cl-) in PM2.5 is?
15. Line 181 to 182, I suggest that the authors add the concentration of mean, maximum and minimum for each season that they monitored.
16. Table 2: The mean value mentioned in general does not match the mean of the other seasons.
17. The authors can segregate the metal concentrations as carcinogenic and non-carcinogenic compounds and can perform some analysis based on that.
18. The conclusion section also needs to be discussed properly. This should include the major results and different analyses, as well as the carry-forward of the research and future perspectives, which need to be mentioned in the conclusion.
