# Supplementary material for: Chemical composition, seasonal variability and source identification of PM2.5 in an urban background site in Medellín—Colombia
Source: PLoS One. 2026 May 27;21(5):e0350267. doi: 10.1371/journal.pone.0350267 (PMC13215534; doi:10.1371/journal.pone.0350267)
Supplement: Attachment — Submitted filename: Rebuttal letter.pdf [file pone.0350267.s003.pdf]

Date: January 10, 2026
Journal: PLOS One
Manuscript ID: [PONE-D-25-58360]
Title: Chemical Composition, Seasonal Variability, and Source Identification of $PM_{2.5}$ at an Urban Background Site in Medellín—Colombia
Authors: Mauricio A. Correa-Ochoa, Miriam Gómez-Marín, Kelly Viviana Patiño-López, Luisa M. Gómez Pelaez, and Santiago A. Franco.

To: The Editorial Board and Reviewers

PLOS One

Subject: Submission of Revised Manuscript and Response to Reviewers' Comments.

Dear Editor and Reviewers,

Thank you very much for the opportunity to revise our manuscript. We would like to express our sincere gratitude to the reviewers for their insightful comments and constructive suggestions, which have significantly helped us improve the quality and clarity of our work.

We have carefully addressed each of the points raised. In the following pages, we provide a point-by-point response to the reviewers' comments. For clarity, the reviewers' comments are presented in **bold**, followed by our detailed responses. Changes made in the revised manuscript have been highlighted using [e.g., track changes / blue text] to facilitate the review process.

General comments on the requested corrections:

Although the previous study "Characteristic chemical profile of PM2.5 particles: a comparative study between two periods. Case study in Medellín, Colombia" https://doi.org/10.3390/su17125380 contains a station contemplated in the present investigation, the previous one corresponds to the estimation of the characteristic profile of PM2.5 particles in two periods in different areas of the Aburrá Valley and nearby. In this study, the focus was on comparing the chemical compositions and the changes found between periods. The focus of the present investigation goes far beyond a temporal comparison, since several key aspects are analyzed, which we will now discuss: the analysis of climatic variability, in which the typical wet and dry periods are evaluated, as well as the biannual precipitation of the city of Medellín. This analysis is further detailed in the paper, since precipitation data were obtained from a meteorological station belonging to the main meteorological data entity of the country. This station is located one kilometer away from the point where the air quality station where PM2.5 samples were taken is located. Another important difference is the receiver model analysis (in this case, principal component analysis), which detected emission sources supported by multivariate statistics and the typical

correlations between pollutants that are tracers of specific sources. Another important difference lies in the analysis of hot spots and back trajectories.

Regarding Kinetics of UO2 dissolution under highly alkaline conditions: Application of a thin film continuous flow-through reactor (https://upcommons.upc.edu/server/api/core/bitstreams/914956f5-d608-4414-a995-c1661105f8e2/content), we understand that there may be similarities, especially in the methodological part; however, this is because the methods are standardized and usually the same steps and descriptions are used to describe the procedures. In this case, we have made sure to improve the methodological section.

We do not wish to manifest changes in our sponsors or upload our procedures to protocols.io, as we have employed standardized methodologies widely used worldwide for the chemical analyses we have performed. We have also checked that all sources in both the preliminary and current versions of the document are correctly referenced, thus acknowledging the work of the scientific community around our topic of study.

We have added all the key documents for the preparation of the study in a ZIP folder, where a Jupyter notebook, programmed in Python, with the databases and algorithms that allow us to obtain the figures and data presented, is found as a fundamental input (Sup. File 1.zip). For this reason, we consider that our document contains all the information necessary for a scientist to replicate our findings. However, if someone wishes to request more information about the study, we offer the possibility of sharing more information upon formal request. We reserve the right of reply, as we leave all replication data available.

We emphasize in this section that our study did not include sensitive information involving restrictions due to ethical and/or legal reasons. With respect to figure 1, we have made changes in it to use images taken from free and open repositories, where it is not necessary to request licenses for use, since they work under the CC BY 4.0 license. To maintain consistency with the new graphic resources, we have updated figures 2 and 2b: the first one becomes figure 3 and we have omitted the second one, since with the new resources added it could be redundant. This resource can be found in our supplementary material. With this, we improved the graphic quality of the paper, as requested by the journal editor. We have removed all figures from the final document and attached them in TIFF format in the document upload platform. In the present study we have not found any suggestions for citations by the reviewers. We have added more information on permits obtained to work at the study site.

Response to Reviewer 1:

We have corrected the spot changes suggested by the reviewer regarding syntax and added the standard deviations to the pollutant concentrations for the overall period and for each of the periods. We have expanded the discussion of traffic emissions relative to internal city emissions and reported emissions per ton per year for this source. We have eliminated the table in which we presented the contribution of the component (PC) to the total mass of the pollutant, as the rigor of these calculations did not meet the requirements of the journal. We have contrasted this source information with the social and environmental characteristics that

appear in this new version. In the introduction section, the reviewer states that the information is based on few previous studies. We refer to the fact that it is complex to find studies that quantify the relationships between pollutants and climate variables. For this reason, we do not make references, since this idea is based on the bibliographic search carried out in the framework of this research. To correct this ambiguity, we have changed the wording to make it clear what we are referring to. In the methodological section, we have added information on the context surrounding the study site.

In the discussion section, we have added more information on biomass burning and included an important part in the structure of the article consisting of hot spot analysis, supported with figures and back trajectory analysis. We leave for a future work the discretization of biomass sources, since in this paper we focus on what happens in the country and, more specifically, in the valley. We have placed Table 1 in the methodology section so that the discretization performed in the sampling and the number of samples taken in each of the periods are clearer.

In the discussion section, more information on biomass burning has been added and an important part of the article consists of hot spot analysis, supported with figures and back trajectory analysis. We leave for future work the discretization of biomass sources, since things happen in the country and, more specifically, in the valley. We have placed Table 1 in the methodology section so that the discretization performed in the sampling and the number of samples taken in each of the periods are clearer. We have expanded the discussion of why dry period 1 had the lowest concentrations of material. Due to the nature of the new plots included, we have employed a new library of plots so that everyone's style is standardized and the document has better aesthetics. We have taken into account the reviewer's suggestions and have added the dates on the x-axes of each of the graphs that require it. As we have done with the biomass, we have added relevant and contrasted information on precipitation periods, which are taken by the main meteorological institute of Colombia and are at a station located two kilometers from the measuring station. Regarding the pandemic issue, we chose not to include this information, since our study period does not cover the confinement extensively. Although it is public knowledge that in the Aburrá Valley the concentrations of particulate matter were at medium-high levels during the confinement, we have considered the relevance of this topic in relation to our research and, for this reason, we have decided not to include this information.

Response to Reviewer 2:

We have also added information on the uncertainties in the quantification methods. We have expanded the methodological information on our quantification techniques; however, we consider that this aspect may become redundant, since we have employed standardized methods widely used in similar studies, so they are usually referenced and known by the community. In addition, we have left references for each of these methods, which consist of extensive documents where the particular procedures for each research need are clearly explained. We also emphasize that these procedures have been carried out by laboratories recognized nationally by the main environmental studies entity in Colombia, which are governed by clearly established technical norms and, in many cases, comply with the standards of U.S. and European agencies. We understand that the internal management of each IDEAM-accredited laboratory may contain confidential protocols, so it is not possible

to accurately describe each procedure. However, we adhere to the standardized protocol reported by the laboratory for the handling, quantification and quality of the reported data. We have added standard deviation data for the major contaminants for each of the periods studied. We have collected FIRMS data (VIIRS J2) to contrast the hot spots of the Colombian region and the Aburrá valley, finding important characteristics that support the arguments made in the first version. Similarly, we have performed an analysis of back trajectories, which we have studied with Hysplit at critical dates. We have also provided information on the city's traffic density and annual emissions, as well as on the city's industrial emissions.

Response to reviewer 3:

1. In many places, the authors used very generic statements that should be replaced with scientific terminology. This needs to be addressed throughout the manuscript.

We have improved the content of the manuscript by improving the wording and using more technical terms throughout the document.

2. The flow of the introduction needs to be re-examined. A few statements contradict others within the introduction and need to be addressed.

We do not consider there to be any contradictory ideas in the document; perhaps they were expressed incorrectly, which led the reviewer to reach this conclusion. We have worked hard to ensure that interpretations like this do not have a place in the current version of the document.

3. The introduction should explain how this study will be useful for further research by researchers or policymakers, and what new information it provides to existing scientific knowledge.

This aspect has been addressed explicitly in the discussion and conclusions, as it is an important aspect, since it is one of the purposes of the study and of science in general. We have added a section to the introduction to make readers aware of the importance of our document.

4. Section 2.1 should include information on local sources such as industries and transport corridors that surround the study area.

We have added more context about the area surrounding the study area.

5. Section 2.2 contains many abbreviations that need to be used consistently throughout the manuscript.

We have reviewed the abbreviations and corrected them where necessary.

6. Sections 2.3.1 to 2.3.3 can be combined into a single paragraph mentioning the methodology and procedure followed. These can be referred to in previous studies conducted by the authors and mentioned here with a reference instead of explaining the procedure again.

Other reviewers have requested a more detailed description of the methods, so we have decided to leave these sections as they were in the first version of the document.

7. The results section seems weak and could benefit from more rigorous scientific discussion. The authors should provide more analysis to illustrate the differences in concentration between the wet 1, wet 2, dry 1 and dry 2 seasons. Interannual changes.

We have strengthened our results by supporting them with new information on hotspots, precipitation and back-trajectories in the study area, and expanded the discussion on what happens in each of the periods of our study.

8. The authors should investigate the different mean and sum concentrations mentioned in the manuscript. There are numerous mismatches in mean and sum values throughout the manuscript.

Very important aspect highlighted by the reviewer. We appreciate the thorough review of the document. We have reanalyzed the information to avoid making this type of error. We have solved the inconsistencies and are working firmly on a single dataset, which is attached to the document and contains the information so that the experimentation can be replicated by anyone with basic knowledge of Python and R Studio.

9. Figures 2 and 3,4 are not properly labelled. Only the unit is written. What do they represent, e.g. mass concentration, and what is being counted? This needs to be updated.

We have significantly improved the presentation of our graphs throughout the document.

10. The discussion sections need significant improvement. The section contains redundant sentences and the information is not discussed scientifically.

We have worked hard to improve the language in our document, and we have discussed and supported the information with our analyses and the data reported by entities such as NOAA and NASA, as well as IDEAM. We have also contrasted the information with solid bibliographic references to widely referenced and published studies in recognised and cutting-edge journals in the field of research.

Specific comments:

1. On line 32, the authors used generic statements instead of specific scientific terminology. For example, 'within cities' could be replaced with 'local sources'.

This has been corrected.

2. Similarly, on line 33, 'emission generated miles away' was replaced with 'long-range transportation of sources'.

We have corrected this.

3. On line 34, PM2.5 is referred to as 'aerosols'. In most cases, aerosols are referred to as two smaller particles.

We have changed this.

4. Lines 41–42 remain as 'deposited'.

We have corrected this.

5. Line 42. The sentence contradicts the previous statement.

We do not consider the sentences to contradict each other; however, we have changed the wording so that the idea is clearer.

6. Lines 60 to 70 could be revised to provide basic information about the study area and the results of previous studies instead of CCN, since the impacts of CCN are more significant with particles in the smaller size range.

We agree with the reviewer's observation, so we have removed the idea related to CCN. Throughout the introduction and discussion, references are made to studies conducted in Medellín and Colombia. We have not delved into this aspect for this specific section.

7. Lines 78 to 79 provide information on how the concentration increased over time in Medellín.

We have discussed this in other sections of the document and do not consider it pertinent to include it in this specific section.

8. Line no. 95: Check one temperature unit.

Correction made.

9. Lines 98 to 100 do not match the information provided in lines 180. Cross-check and correct.

We have reviewed the additional information and verified its consistency. We have moved Table 1 to the Methodology section to make it more consistent with what we want to show.

10. If possible, replace Figure 1 with an image showing the surrounding Ares road network instead of the DEM map.

We have replaced Figure 1 in accordance with the style of the journal and the reviewer's suggestions.

11. On line 115, the authors should clarify whether the filter papers were desiccated before and after the measurement to remove moisture. Moisture content plays a major role in the chemical composition of the collected sample. If not preserved properly, the composition of the measured pollutants will change.

We have specified our procedures; however, we would like to emphasise that these procedures were carried out by a laboratory that follows standardised procedures issued by European and US environmental agencies, as well as technical standards issued by Colombia's leading environmental studies institute.

12. Line 120 mentions that 112 samples were collected, but Table 1 shows that the total number of samples collected is 110. This needs to be corrected.

We have corrected this information and thank the reviewer for pointing out this inconsistency.

13. Inconsistent verb tenses. Use the past tense only. Line no. 128. The samples are then stored at -20 ± 3 °C until chemical characterisation.

We have corrected line no. 14. Line no. 142: What is the concentration of water-soluble ions (K+, Na+, Mg2+, Ca2+, SO42-, NO3-, F- and Cl-) in PM2.5?
We have added this to the discussion.

15. From lines 181 to 182, I suggest that the authors add the mean, maximum and minimum concentrations for each monitored season.

16. Table 2: The mean value mentioned does not generally match the mean of the other seasons.

We accept the reviewer's suggestion and have added the table.

17. The authors could categorise the metal concentrations as carcinogenic or non-carcinogenic compounds and perform an analysis based on this categorisation.

We consider that we have a fairly comprehensive analysis, and this information will be introduced in the text without delving too deeply into the implications of these results for the topic, as this would divert attention from the focus of our study.

18. The conclusion section also needs to be discussed properly. This should include the main results and different analyses, as well as the continuation of the research and future perspectives, which must be mentioned in the conclusion.

We have improved the conclusions section by using more technical language and aligning it with the results of the analyses introduced in the current version.

We believe that these revisions have strengthened the manuscript and addressed all the concerns raised. We hope that the revised version is now suitable for publication in PLOS One.

Thank you for your time and consideration.

Sincerely,

Mauricio A. Correa-Ochoa Corresponding Author

G-LIMA, Universidad de Antioquia

mandres.correa@udea.edu.co
